# NVX-CoV2373 vaccination induces functional SARS-CoV-2–specific CD4^+^ and CD8^+^ T cell responses

**DOI:** 10.1172/JCI160898

**Published:** 2022-10-03

**Authors:** Carolyn Rydyznski Moderbacher, Christina Kim, Jose Mateus, Joyce Plested, Mingzhu Zhu, Shane Cloney-Clark, Daniela Weiskopf, Alessandro Sette, Louis Fries, Gregory Glenn, Shane Crotty

**Affiliations:** 1 Center for Infectious Disease and Vaccine Research, La Jolla Institute for Immunology (LJI), La Jolla, California, USA.; 2 Novavax Inc., Gaithersburg, Maryland, USA.; 3 Department of Medicine, Division of Infectious Diseases and Global Public Health, UCSD, La Jolla, California, USA.

**Keywords:** Immunology, Adaptive immunity, Cellular immune response, T cells

## Abstract

NVX-CoV2373 is an adjuvanted recombinant full-length SARS-CoV-2 spike trimer protein vaccine demonstrated to be protective against COVID-19 in efficacy trials. Here we demonstrate that vaccinated individuals made CD4^+^ T cell responses after 1 and 2 doses of NVX-CoV2373, and a subset of individuals made CD8^+^ T cell responses. Characterization of the vaccine-elicited CD8^+^ T cells demonstrated IFN-γ production. Characterization of the vaccine-elicited CD4^+^ T cells revealed both circulating T follicular helper (cTfh) cells and Th1 cells (IFN-γ^+^, TNF-α^+^, and IL-2^+^) were detectable within 7 days of the primary immunization. Spike-specific CD4^+^ T cells were correlated with the magnitude of the later SARS-CoV-2–neutralizing antibody titers, indicating that robust generation of CD4^+^ T cells, capable of supporting humoral immune responses, may be a key characteristic of NVX-CoV2373 that utilizes Matrix-M adjuvant.

## Introduction

The ongoing COVID-19 pandemic caused by severe acute respiratory syndrome coronavirus 2 (SARS-CoV-2) has caused immense morbidity and mortality. This global crisis has been met with an unprecedented surge in vaccine development and implementation. The various COVID-19 vaccines that have been approved for use in humans have vastly improved health outcomes to COVID-19 disease, but the important hurdle of providing COVID-19 vaccines to every person in the world remains.

NVX-CoV2373 by Novavax is composed of recombinant full-length, stabilized prefusion spike protein homotrimers that form approximately 30-nm nanoparticles based on hydrophobic interaction with a central polysorbate-80 micelle (1). The vaccine antigen, based on the ancestral Wuhan-Hu-1 strain of SARS-CoV-2, is formulated with Matrix-M adjuvant (2). The vaccine is stable at 2°C to 8°C, making it amenable for deployment to regions where refrigeration is limited, increasing vaccine access for populations where other COVID-19 vaccines are not easily distributed or stored. NVX-CoV2373 has demonstrated efficacy in phase IIb and III clinical trials in the United Kingdom (3), South Africa (4), and a large phase III trial in the United States and Mexico (5). NVX-CoV2373 has emergency use authorization in multiple countries around the globe (6). A more detailed understanding of the immune response to this vaccine is important for the ongoing global efforts to combat COVID-19 infections and deaths, especially in the wake of new and emerging variants (7). Here, we set out to determine the T cell response to the NVX-CoV2373 vaccine, utilizing samples from individuals enrolled in a phase I/IIa clinical trial who received 5 μg NVX-CoV2373 protein adjuvanted with Matrix-M (1, 8).

The presence of T cells capable of recognizing SARS-CoV-2 epitopes in SARS-CoV-2–uninfected individuals was first reported in May of 2020 (9–13). These cross-reactive T cells were later confirmed to be memory T cells, some of which were generated in response to infections with commonly circulating human coronaviruses (HCoVs OC43, 229E, NL63, and HKU1) and capable of cross-reacting with shared SARS-CoV-2 epitopes (11, 13, 14). This finding led to speculation that these cells could have an impact on an individual’s immune response to SARS-CoV-2 infection. Theories proposed that these preexisting T cells could be beneficial or detrimental for immunity to SARS-CoV-2 (15). However, at the time it was unknown what biological effect these cross-reactive memory CD4^+^ T cells might have in the context of COVID-19 vaccination. Therefore, in addition to measuring human CD4^+^ and CD8^+^ T cell responses to NVX-CoV2373, we explored the effect of cross-reactive CD4^+^ T cells on the human immune response to the NVX-CoV2373 vaccine.

## Results

### NVX-CoV2373 induces SARS-CoV-2–specific CD4^+^ T cells.

**Figure 1:**
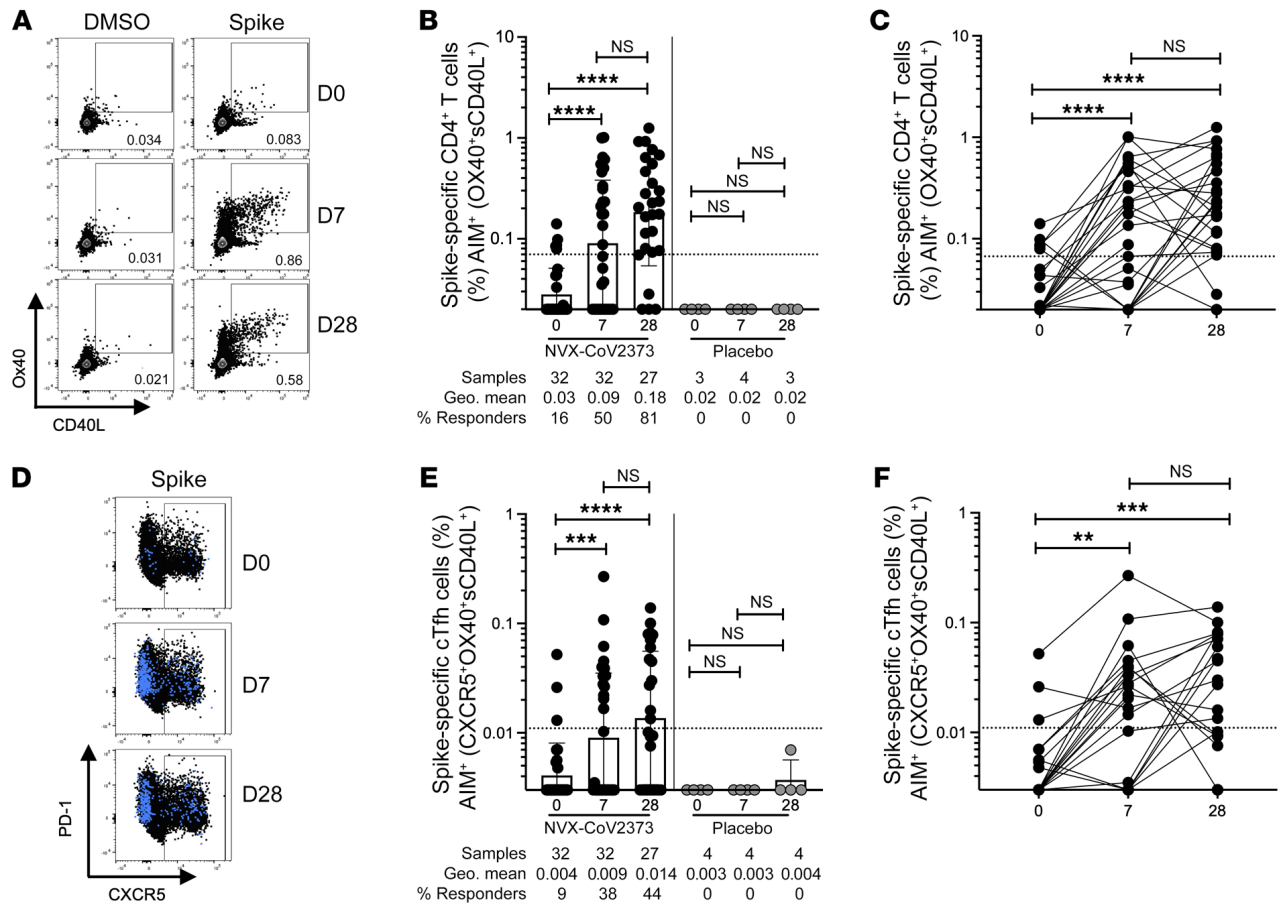
Spike-specific CD4^+^ T cells following NVX-CoV2373 vaccination. (**A**) Representative FACS plots of AIM^+^ (sCD40L^+^OX40^+^) CD4^+^ T cells on day 0 (D0), D7, and D28 after vaccination. (**B** and **C**) Spike-specific AIM^+^ CD4^+^ T cell responses in vaccinees (black dots) and placebo controls (gray dots). The same data are graphed as grouped (**B**) and paired comparisons (**C**). (**D**) Representative FACS plots of AIM^+^CXCR5^+^ CD4^+^ cTfh cells (blue dots are AIM^+^ CD4^+^ T cells overlaid on total CD4^+^ T cells in black). (**E** and **F**) Spike-specific AIM^+^ cTfh cell responses in vaccinees and placebo controls. The same data are graphed as grouped (**E**) and paired comparisons (**F**). Dotted line indicates limit of quantitation (LOQ) for the assay, and was calculated as the geometric mean of all sample DMSO wells multiplied by the geometric SD. Percentage responders are calculated as responses ≥ LOQ divided by the total samples in the group. Paired data were analyzed by Wilcoxon’s signed-rank test. Data shown as geometric mean ± geometric SD. **P < 0.01; ***P < 0.001; ****P < 0.0001.

Peripheral blood mononuclear cells (PBMCs) from 27 volunteers immunized with 5 μg of NVX-CoV2373 on days 0 and 21, 5 volunteers immunized with 5 μg of NVX-CoV2373 on day 0 and placebo on day 21, and 4 recipients of placebo, were isolated from blood samples taken on days 0, 7, and 28. The 5 volunteers that received the placebo dose on day 21 were only included for analysis on days 0 and 7 in all analyses reported in this study. SARS-CoV-2 spike–specific CD4^+^ T cells were measured by activation-induced marker (AIM) assay (surface CD40L^+^ [sCD40L^+^] and OX40^+^; Figure 1A and Supplemental Figure 1A; supplemental material available online with this article; https://doi.org/10.1172/JCI160898DS1). On day 0, 16% of donors (5 of 32) had detectable SARS-CoV-2 spike–specific CD4^+^ T cells, detectable above the limit of quantitation (LOQ) for the assay, indicative of preexisting T cell memory (Figure 1, B and C). Notably, by day 7 after the first immunization, 50% of donors (16 of 32) had developed spike-specific CD4^+^ T cell responses (Figure 1, B and C). A majority of donors (81%, 22 of 27) exhibited high levels of SARS-CoV-2 spike–specific CD4^+^ T cells 7 days after the second immunization (28 days after the first immunization; Figure 1, B and C). There was no significant difference in the magnitude of the antigen-specific CD4^+^ T cell response when comparing 7 days after the first immunization with 7 days after the second immunization (Figure 1, B and C). However, the proportion of individuals mounting a detectable SARS-CoV-2 spike response after the second immunization was significantly increased relative to after the first immunization (Fisher’s exact test, *P =* 0.015). CD4^+^ T cell response results were comparable when using the OX40^+^4-1BB^+^ AIM assay (Supplemental Figure 1B), or when calculated by stimulation index (Supplemental Figure 1E). To verify that the spike T cell response was vaccine specific, a peptide megapool (MP) containing predicted SARS-CoV-2 class II epitopes spanning the entire SARS-CoV-2 proteome sans spike (hereafter referred to as “non-spike”), as well as a CMV MP containing class I and II epitopes, were run in parallel for a subset of donors. There were no significant differences in the CD4^+^ T cell response to non-spike or CMV MPs across the study time points, validating the NVX-CoV2373 vaccine spike–specific CD4^+^ T cell response detected (Supplemental Figure 1, C and D).

Functionalities of NVX-CoV2373–induced CD4^+^ T cell responses were assessed by identifying spike-specific circulating follicular helper T (cTfh) cells and by intracellular cytokine staining (ICS) of spike-specific CD4^+^ T cells. Tfh cells are crucial for antibody responses following infection or vaccination. Similar to total AIM^+^ (sCD40L^+^OX40^+^) CD4^+^ T cells, AIM^+^ cTfh cells (CXCR5^+^AIM^+^ CD4^+^ T cells) were significantly increased relative to day 0 in 36% of vaccinees (12 of 32) 7 days after the first immunization and 44% (12 of 27) 7 days after the second immunization (Figure 1, D–F). There was no significant difference in the magnitude of spike-specific cTfh cells after the first compared to the second immunization, or the proportion of individuals that developed spike-specific cTfh cells (Figure 1, E and F; Fisher’s exact test, *P =* 0.61).

**Figure 2:**
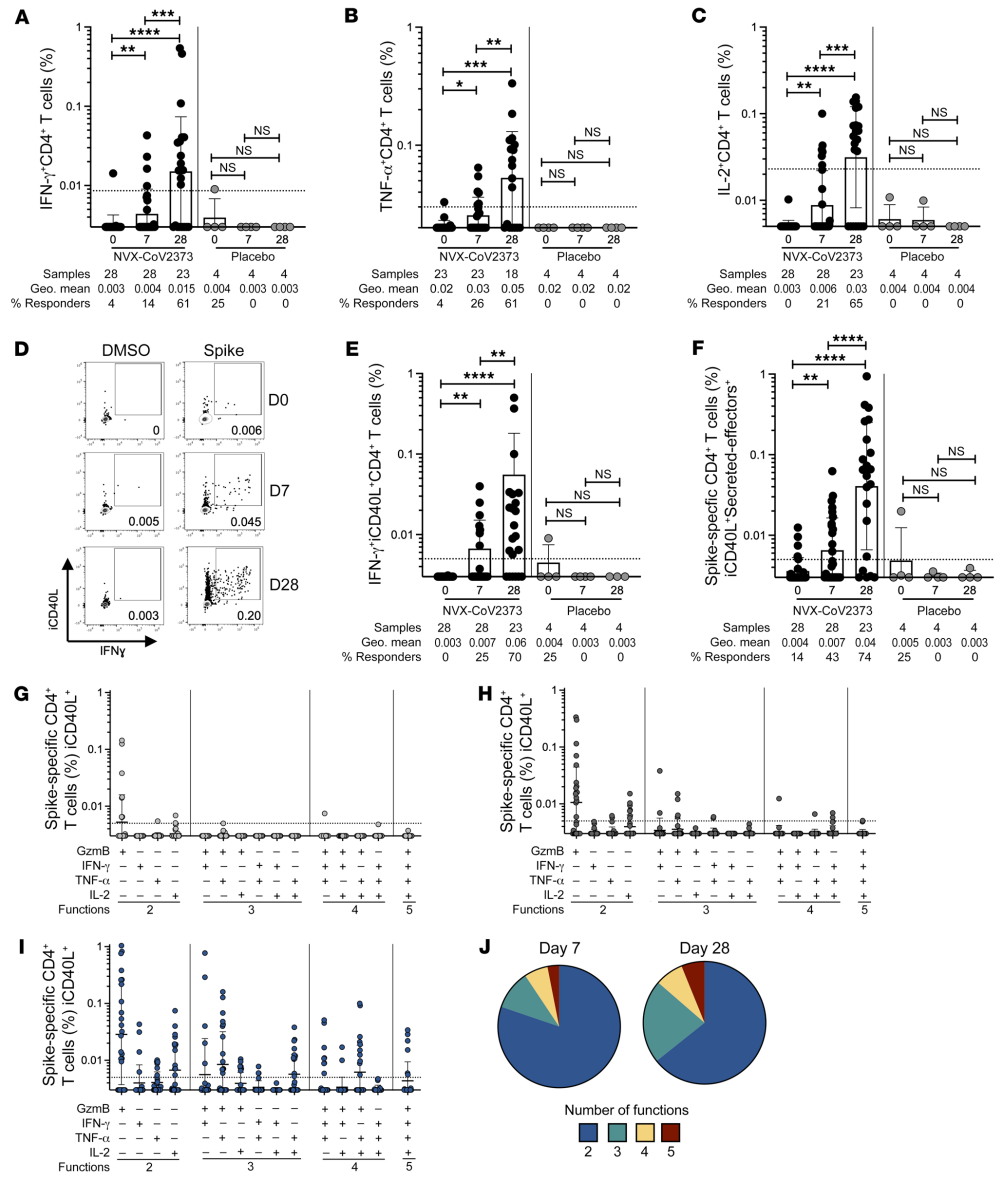
Cytokine-producing spike-specific CD4^+^ T cell responses following NVX-CoV2373 vaccination. Proportion of (**A**) IFN-γ^+^, (**B**) TNF-α^+^, and (**C**) IL-2^+^ spike-specific CD4^+^ T cells detected following peptide stimulation. (**D**) Representative FACS plots and (**E**) proportion of IFN-γ^+^ intracellular CD40L^+^ (iCD40L^+^) responses in spike-specific CD4^+^ T cells on days 0, 7, and 28 after vaccination. (**F**) Proportion of spike-specific CD4^+^ T cells expressing iCD40L and producing IFN-γ, TNF-α, IL-2, or GzmB (“secreted-effector^+^”). Predominant multifunctional profiles of spike-specific CD4^+^ T cells with 1, 2, 3, 4, or 5 functions were analyzed on (**G**) day 0 (D0), (**H**) D7, and (**I**) D28 after vaccination. (**J**) Pie charts depicting the proportion of spike-specific CD4^+^ T cells exhibiting 2, 3, 4, or 5 functions on day 7 and day 28 after immunization. Functionality is defined as a cell expressing iCD40L and any combination of IFN-γ, TNF-α, IL-2, or GzmB. Dotted line indicates LOQ for the assay, and was calculated as the geometric mean of all sample DMSO wells multiplied by the geometric SD. Percentage responders was calculated as responses ≥ LOQ divided by the total samples in the group. Paired data were analyzed by Wilcoxon’s signed-rank test. Data shown as geometric mean ± geometric SD. *P < 0.05; **P < 0.01; ***P < 0.001; ****P < 0.0001.

Cytokine production by NVX-CoV2373–induced spike-specific CD4^+^ T cells was assessed via ICS. Significant increases in spike-specific IFN-γ^+^, TNF-α^+^, and IL-2^+^ CD4^+^ T cells were observed 7 days after the first immunization (Figure 2, A–C). IFN-γ–, TNF-α–, and IL-2–secreting CD4^+^ T cell frequencies were further increased after the second immunization (Figure 2, A–C). IL-17A^+^, IL-4^+^, or IL-10^+^ spike-specific cells were not detected (Supplemental Figure 2, A–C). Spike-specific IFN-γ^+^ intracellular CD40L^+^ (iCD40L^+^) double-positive CD4^+^ T cells were significantly increased after both the first and second immunizations relative to baseline (Figure 2, D and E), consistent with the IFN-γ^+^ single gating (Figure 2A), and included the vast majority of the IFN-γ^+^ cells (Supplemental Figure 2A). The total cytokine^+^ CD4^+^ T cell response (sum of iCD40L^+^ cells expressing granzyme B [GzmB], IFN-γ, TNF-α, or IL-2) was significantly increased after the first and after the second immunization relative to baseline (Figure 2F). The overall cytokine profile was indicative of a Th1 response, appropriate for antiviral immunity. Polyfunctional CD4^+^ T cell cytokine responses were also induced by NVX-CoV2373 vaccination. The proportion of spike-specific CD4^+^ T cells exhibiting 2, 3, 4, or 5 functions (iCD40L, GzmB, IFN-γ, TNF-α, IL-2) was increased after the first and second immunization relative to baseline (Figure 2, G–I), and a larger proportion of spike-specific CD4^+^ T cells exhibited 3 to 5 functions 1 week after the second immunization (35%) relative to spike-specific CD4^+^ T cells after the first immunization (19%) (Figure 2J and Supplemental Figure 2F). SARS-CoV-2 non-spike IFN-γ^+^iCD40L^+^ CD4^+^ T cell frequencies remained unchanged after immunization, as expected (Supplemental Figure 2E).

cTfh cytokine responses were measured in a subset of donors and their cytokine profile largely mirrored the total CD4^+^ T cell response, with an increase in IFN-γ^+^, TNF-α^+^, and IL-2^+^ cTfh cells detectable 1 week after the second immunization and minimal expression of Th2 cytokines such as IL-4 (Supplemental Figure 2, G–J). Additionally, CD4^+^ T cell AIM and cytokine responses to the Omicron variant spike were comparable to ancestral SARS-2 spike recognition (Supplemental Figure 3, A–E). These results are consistent with a previous study from our group that showed CD4^+^ T cell epitope recognition was highly conserved across SARS-CoV-2 variants as opposed to neutralizing antibody responses (16). Together, these data suggest that the NVX-CoV2373 vaccine induces multifunctional spike-specific CD4^+^ T cells composed of classical Th1 T cells and cTfh cells necessary for supporting antiviral and antibody responses and recognition of diverse variants.

### NVX-CoV2373 induces SARS-CoV-2–specific CD8^+^ T cells.

**Figure 3:**
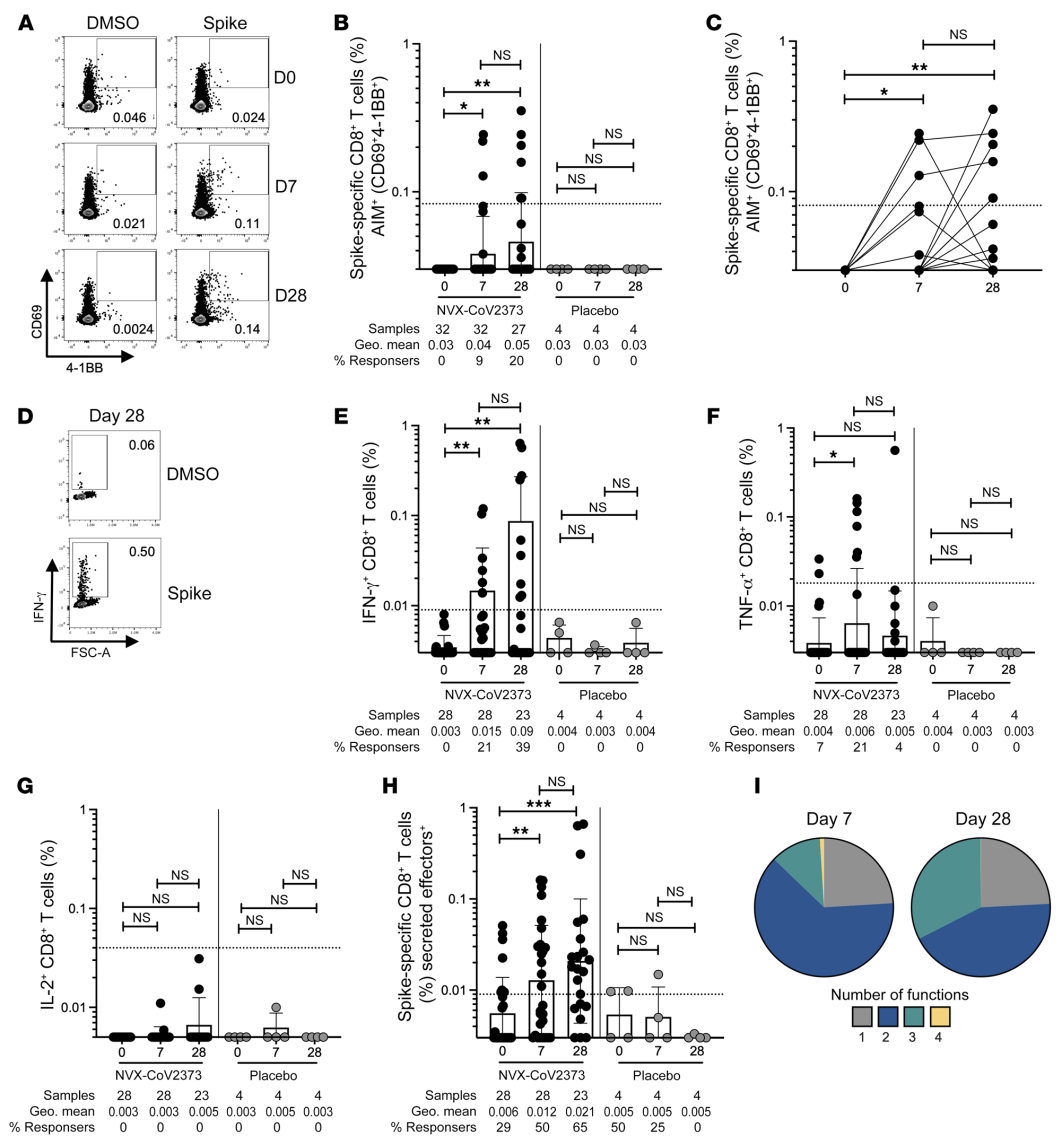
Spike-specific CD8^+^ T cells are induced following NVX-CoV2373 vaccination. (**A**) Representative FACS plots of AIM^+^ (CD69^+^4-1BB^+^) CD8^+^ T cells on day 0 (D0), D7, and D28 after vaccination. (**B** and **C**) Spike-specific AIM^+^ CD8^+^ T cells responses in vaccinees (black dots), placebo controls (gray dots), and convalescent COVID-19 donors (blue dots). (**D**) Representative FACS plots of IFN-γ responses in total spike-specific CD8^+^ T cells. IFN-γ^+^ (**E**), TNF-α^+^ (**F**), and IL-2^+^ (**G**) spike-specific CD8^+^ T cell responses in vaccinees, placebo controls, and convalescent COVID-19 controls. (**H**) Secreted-effector^+^ spike-specific CD8^+^ T cells (sum of CD8^+^ T cells expressing any combination of IFN-γ, TNF-α, IL-2, or GzmB, excluding GzmB single positives). (**I**) Pie charts depicting the proportion of spike-specific CD8^+^ T cells exhibiting 1, 2, 3, or 4 functions on day 7 and day 28 after immunization. Functionality was defined as a cell expressing any combination of IFN-γ, TNF-α, IL-2, or GzmB, excluding GzmB single positives. Dotted line indicates limit of sensitivity for the assay, and was calculated as the geometric mean of all sample DMSO wells multiplied by the geometric SD factor. Percentage responders was calculated as responses ≥ LOQ divided by the total samples in the group. Paired data were analyzed by Wilcoxon’s signed-rank test. Data shown as geometric mean ± geometric SD. *P < 0.05; **P < 0.01; ***P < 0.001.

Spike-specific CD8^+^ T cell responses following the first and second immunizations with the NVX-CoV2373 vaccine were tested by AIM and ICS assays (Figure 3A, Supplemental Figure 4A, and Supplemental Figure 5A). No donors had detectable CD8^+^ T cells by AIM at baseline (CD69^+^4-1BB^+^; Figure 3, A–C). Seven days after the first immunization, 9% of donors (3 of 32 donors) had a modest spike-specific CD8^+^ T cell response detected by AIM. One-week after the second immunization, 20% of donors had developed a spike-specific CD8^+^ T cell response (5 of 27 donors; Figure 3, A–C). Stimulation indices similarly showed significant NVX-CoV2373 vaccine–induced CD8^+^ T cell AIM^+^ responses after the first and second immunization (Supplemental Figure 4D). There was no significant difference in the amplitude of CD8^+^ T cell responses between post–first- and post–second-immunization time points (Figure 3C; Fisher’s exact test, *P =* 0.5), but the majority of donors who had developed CD8^+^ T cell responses 7 days after the first immunization retained these responses after the second immunization, and were joined by an additional cohort of CD8^+^ responders after the second dose. There was no difference in the CD8^+^ T cell frequency in response to SARS-CoV-2 non-spike or CMV MPs across all time points assessed, as expected (Supplemental Figure 4, B and C).

SARS-CoV-2 spike–specific CD8^+^ T cell functionality was assessed via ICS. Spike-specific IFN-γ^+^ CD8^+^ T cells were detected in a small subset of donors after the first immunization (11%, 3 of 28 donors) and increased after the second immunization (26%, 6 of 23 donors; Figure 3, D and E). Similar increases in IFN-γ^+^ CD8^+^ T cells after the first and second immunizations were also observed when the data were plotted as stimulation index (Supplemental Figure 5B). TNF-α was modestly increased after the first (21% 6 of 28 donors), but not second immunization (Figure 3F). IL-2^+^ single-positive CD8^+^ T cells were not detected (Figure 3G). The total cytokine^+^ CD8^+^ T cell response (sum of CD8^+^ T cells expressing any combination of IFN-γ, TNF-α, IL-2, or GzmB, excluding GzmB single positives), was significantly increased after both the first and second immunizations relative to baseline (Figure 3H). Polyfunctional CD8^+^ T cell cytokine responses were also induced by NVX-CoV2373 vaccination. The proportion of spike-specific CD8^+^ T cells exhibiting 2, 3, or 4 functions (GzmB, IFN-γ, TNF-α, IL-2) was increased after the first and second immunization relative to baseline (Supplemental Figure 5, C–F). The proportion of spike-specific CD8^+^ T cells positive for 3 functions was increased after the second immunization (29%) relative to the first immunization (9%) (Figure 3I and Supplemental Figure 5F). In sum, spike-specific CD8^+^ T cell responses were induced in some individuals following NVX-CoV2373 vaccination, as measured by 2 experimental approaches.

### Relationship between T cell and antibody responses following NVX-CoV2373 vaccination.

**Figure 4:**
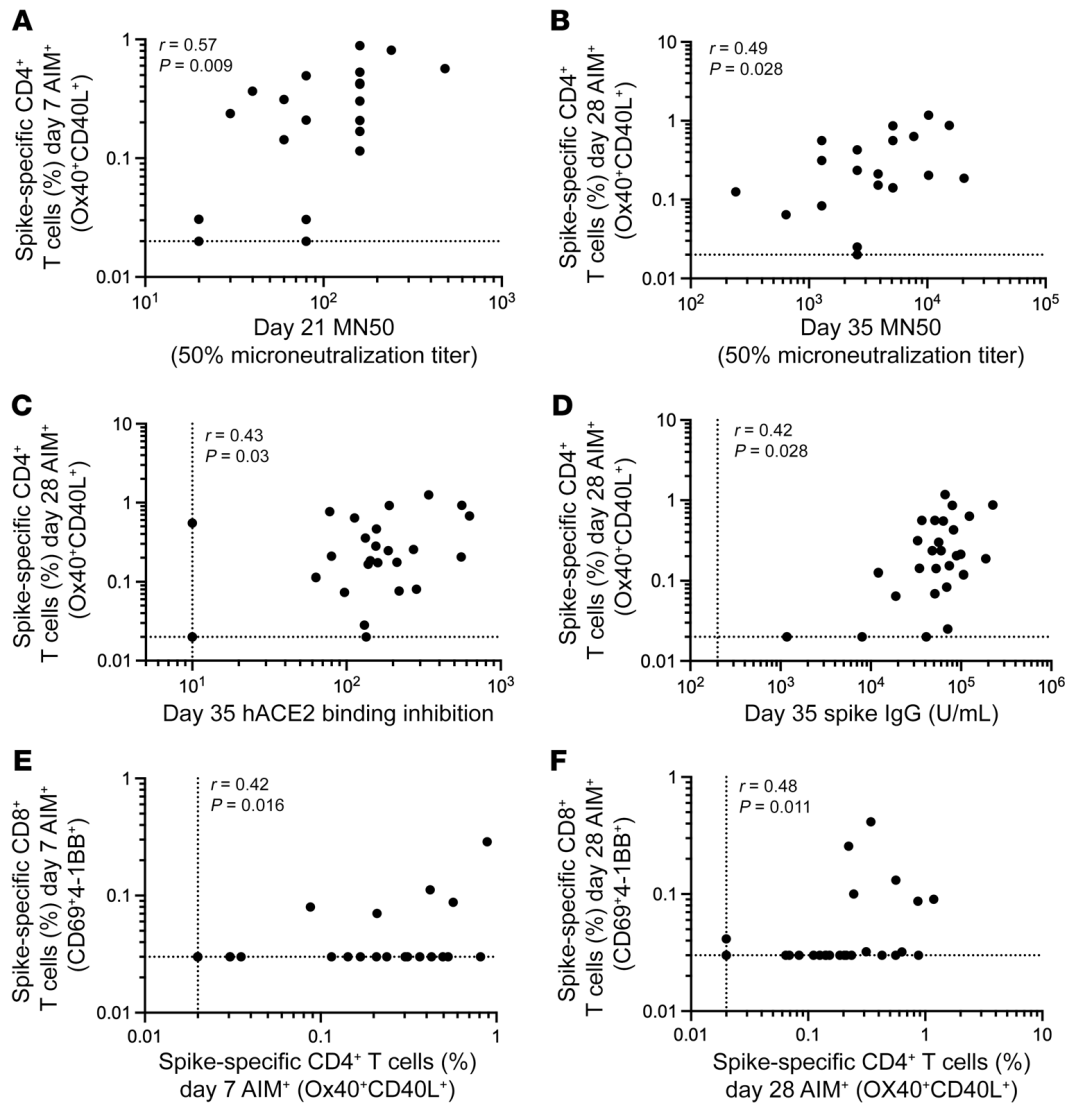
Association between T cell and antibody responses following NVX-CoV2373 vaccination. Correlation between day 7 SARS-CoV-2 spike–specific AIM^+^ CD4^+^ T cells and (**A**) day 21 microneutralization titers or (**B**) D28 SARS-CoV-2 spike–specific AIM^+^ CD4^+^ T cells and day 35 neutralization titers. Correlation between day 28 SARS-CoV-2 spike–specific AIM^+^ CD4^+^ T cells and (**C**) day 35 hACE2 binding inhibition or (**D**) day 35 spike IgG. Correlation between spike-specific AIM^+^ CD4^+^ cells and AIM^+^ CD8^+^ cells on (**E**) day 7 and (**F**) day 28. Data were analyzed by Spearman’s correlation.

Samples were analyzed for anti-spike IgG titers via ELISA and SARS-CoV-2 neutralization activity using both microneutralization and hACE2 binding inhibition assays (Supplemental Figure 6, A–C). Anti-spike IgG and neutralizing antibodies were induced following the first immunization and further enhanced upon the second immunization (Supplemental Figure 6, A–C). Spike-specific CD4^+^ T cells and antibody responses were assessed for relationships. Spike-specific (AIM^+^) CD4^+^ T cells did not significantly correlate with anti-spike IgG titers following the first immunization, but were significantly associated following the second immunization (Figure 4D and Supplemental Figure 6D). However, AIM^+^ CD4^+^ T cells were significantly associated with neutralizing antibodies after both the first and second immunization (Figure 4, A–C). Spike-specific cTfh cell frequencies did not measurably correlate with anti-spike IgG or neutralizing antibodies (Supplemental Figure 6, E–H). This was also true when examining CXCR3- and CCR6-expressing cTfh cells (Supplemental Figure 7, A–E), in contrast to associations between cTfh cell subsets and antibody responses observed after SARS-CoV-2 infection (17–19). AIM^+^ CD4^+^ T cell and AIM^+^ CD8^+^ T cell responses were significantly correlated at both 7 days after the first and 7 days after the second immunization (Figure 4, D and E). Together, these data demonstrate the ability of the NVX-CoV2373 vaccine to elicit antibody, CD4^+^ T cell, and CD8^+^ T cell responses against SARS-CoV-2 infection.

### Preexisting T cell immunity did not impact NVX-CoV2373 vaccine responses.

**Figure 5:**
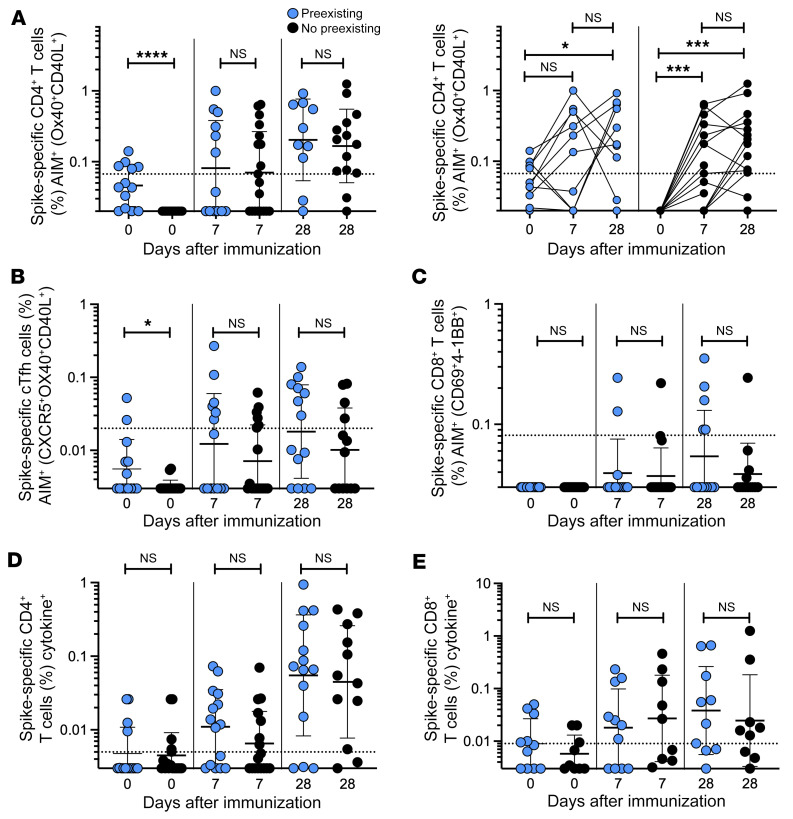
Preexisting T cell immunity does not impact NVX-CoV2373 vaccine responses. (**A**) Spike-specific AIM^+^ CD4^+^ T cells at baseline and after vaccination grouped according to donors with detectable AIM^+^ CD4^+^ T cell responses on day 0 (blue dots) or no detectable AIM^+^ CD4^+^ T cell responses on day 0 (black dots). Effect of preexisting AIM^+^ CD4^+^ T cells on (**B**) spike-specific AIM^+^ cTfh cells, (**C**) spike-specific AIM^+^ CD8^+^ T cells, (**D**) total spike-specific CD4^+^ T cells and (**E**) spike-specific CD8^+^ T cell cytokine production. Dotted line indicates limit of sensitivity for the assay, and was calculated as the geometric mean of all sample DMSO wells multiplied by the geometric SD. Data were analyzed by Mann-Whitney test. Data shown as geometric mean ± geometric SD. *P < 0.05; ***P < 0.001; ****P < 0.0001.

Within the cohort, 10 donors had detectable SARS-CoV-2 spike–specific CD4^+^ T cell responses at baseline (Figure 5A). These donors self-reported no previous SARS-CoV-2 infection or vaccination and were recruited in a time frame when local SARS-CoV-2 seroprevalence was very low. Therefore, these were likely cross-reactive memory T cells, induced via infection with endemic coronaviruses or other infections. Whether such preexisting cross-reactive cells contribute to, detract from, or have no influence on COVID-19 vaccine responses was undetermined. Thus, we explored this topic in the cohort of NVX-CoV2373 vaccinees. Donors were separated into 2 groups based on the presence or absence of cross-reactive T cells at baseline (AIM^+^ CD4^+^ T cell response above the limit of detection [LOD] for the assay [0.02%]). There was no significant difference in the magnitude or frequency of the spike-specific (AIM^+^) CD4^+^ T cell response after the first or second immunization between donors with and without preexisting cross-reactive memory CD4^+^ T cells (Figure 5A). Similarly, we observed no significant differences between the magnitude of AIM^+^ cTfh CD4^+^ T cells or AIM^+^ CD8^+^ T cells based on preexisting cross-reactive memory CD4^+^ T cells (Figure 5, B and C). Individuals with cross-reactive AIM^+^ CD4^+^ T cells also did not exhibit any significant differences in the magnitude of spike-specific cytokine^+^ CD4^+^ or CD8^+^ T cells (Figure 5, D and E). These results were similar when we employed a more rigorous threshold for preexisting T cell immunity, assessing samples with an AIM^+^ CD4^+^ T cell response above the LOQ for the assay (0.067%) (Supplemental Figure 8, A–E). Therefore, individuals with cross-reactive memory CD4^+^ T cells do not appear to be more or less likely to make a stronger immune CD4^+^ T cell response following 2 doses of the NVX-CoV2373 vaccine, although this conclusion is based on limited data.

## Discussion

Understanding COVID-19 vaccine–induced immunity is crucial for implementing immunization regimens that can be used to mitigate the ongoing SARS-CoV-2 pandemic in various regions of the world and diverse populations. Samples from 32 healthy adults were analyzed to determine cellular immune responses to the protein-based COVID-19 vaccine, NVX-CoV2373. Immunization with NVX-CoV2373 induced a SARS-CoV-2 spike–specific CD4^+^ T cell response as early as 7 days after the first immunization, characterized by a substantial cTfh population, as well as polyfunctional cytokine-producing cells dominated by those producing IFN-γ, TNF-α, and IL-2. Notably, NVX-CoV2373 immunization also promoted modest CD8^+^ T cell responses in a subset of donors. CD4^+^ and CD8^+^ T cell responses correlated with each other after both the first and second immunizations, and total AIM^+^ CD4^+^ T cells correlated with neutralizing antibodies.

A combination of AIM assays and ICS were used to characterize SARS-CoV-2 spike–specific CD4^+^ and CD8^+^ T cells induced by NVX-CoV2373 vaccination. Spike-specific CD4^+^ T cells, as measured by AIM assay, could be detected as early as 7 days after the first immunization. This rapid induction of robust spike-specific CD4^+^ T cell responses could be a result of increased immunogenicity from the Matrix-M adjuvant in the NVX-CoV2373 vaccine. Similar to the total spike-specific CD4^+^ T cell response, NVX-CoV2373 also induced a strong cTfh cell response 7 days after the first immunization. A rapid and sustained cTfh response likely supports a more productive antibody response. We did not observe an increase in the magnitude of the T cell response between the post–first-immunization and post–second-immunization time points (20, 21).

Functional cytokine profiles of spike-specific CD4^+^ T cells and cTfh cells revealed Th1 cytokines produced by the CD4^+^ T cell response induced by NVX-CoV2373 vaccination, including IFN-γ, TNF-α, and IL-2, with minimal IL-10 or Th2 or Th17 cytokine profiles observed. Overall, the cytokine data indicate that the vaccine induces a Th1 CD4^+^ T cell response, consistent with earlier reports (1), and mirroring a Th1 bias in the presence of Matrix-M that has been reported in several animal models (2, 20, 21). The cytokine responses increased after the second immunization, with an increase in polyfunctionality continuing after the second dose of vaccine, indicating further polarization of the CD4^+^ T cell response.

In contrast to live-attenuated or inactivated vaccines, protein-based vaccines have historically proven to be poor inducers of CD8^+^ T cell responses in humans (22–24). However, the data presented here indicate that the NVX-CoV2373 vaccine does induce modest spike-specific CD8^+^ T cell responses in a subset of individuals. Spike-specific CD8^+^ T cells were identified by AIM. ICS assays showed similar results, with IFN-γ^+^ CD8^+^ T cells detected after both the first and second immunizations. CD8^+^ T cell responses were less polyfunctional than CD4^+^ T cell responses to NVX-CoV2373 vaccination, but several donors did develop multifunctional CD8^+^ T cell responses following their first or second dose of NVX-CoV2373. Spike-specific CD4^+^ T cell responses correlated with CD8^+^ T cell responses, suggesting that the CD8^+^ T cell response observed may depend on the CD4^+^ T cell response. Additional studies with a larger cohort of vaccinees including seropositive and seronegative individuals are warranted to validate these findings. The Matrix-M adjuvant may be more potent than most adjuvants at inducing CD8^+^ T cell priming, or possibly recalling cross-reactive memory T cells. Amphiphilic saponins, like those present in Matrix-M, are reported to destabilize the endosomal/lysosomal membrane and facilitate entry of antigens into the cytoplasm, a first step in cytosolic catabolism and subsequent presentation in the context of MHC class I to initiate CD8^+^ T cell responses (21).

Cross-reactive CD4^+^ T cells, which antedate SARS-CoV-2 or SARS-CoV-2 vaccine exposure and arise in part from endemic beta- and alpha-coronavirus exposure, are capable of recognizing SARS-CoV-2 epitopes, and have been of substantial interest (9, 11–15, 23–27). One hypothesis suggests cross-reactive T cells could amplify the vaccine response, while others have posited that these cells could largely comprise T cells with relatively low affinity for SARS-CoV-2 and might inhibit higher-affinity T cell clones from participating in the immune response (15). Within this cohort, we identified 10 donors who had spike-specific AIM^+^ CD4^+^ T cells at baseline. We identified no effect of preexisting cross-reactive memory CD4^+^ T cells at baseline on subsequent SARS-CoV-2 spike–specific T cell responses to vaccination with NVX-CoV2373. This is in contrast to reports examining cross-reactive memory CD4^+^ T cells in the context of mRNA COVID-19 vaccines (17, 26). One explanation for this difference between studies could be differences between protein and mRNA vaccine mechanisms of priming of T cell and B cell responses in vivo. Future studies directly comparing preexisting T cells across different vaccine platforms will be necessary to elucidate any role for these cells in vaccine responses.

Spike-specific CD4^+^ T cells induced by NVX-CoV2373 vaccination correlated with SARS-CoV-2 neutralizing titers. While Tfh cells are highly likely to be the CD4^+^ T cell subset responsible for B cell help and neutralizing antibody development after NVX-CoV2373 immunization, spike-specific cTfh cells did not show a statistically significant correlation with neutralizing titers. This could simply be due to sample size, or the greater difficulty in quantifying antigen-specific cTfh cells in blood. Additionally, by directly observing germinal center Tfh cells in lymph nodes of vaccinated individuals, it was observed that spike-specific Tfh cell frequencies in lymph nodes correlated with germinal center responses and neutralizing antibodies, but cTfh cell frequencies in the blood did not (28). Lastly, a markedly enhanced antibody response observed after the first dose of NVX-CoV2373 in baseline seropositives who were previously exposed to pandemic SARS-CoV-2 (29) is consistent with memory CD4^+^ T cells and B cells against spike being recalled by NVX-CoV2373 immunization.

Together, these data support the idea that the NVX-CoV2373 vaccine induces a complex immune response consisting of robust and polyfunctional CD4^+^ T cells producing Th1-type cytokines and a rapid cTfh cell response capable of supporting a substantial neutralizing antibody response, as well as a modest CD8^+^ T cell response in a subset of donors. Overall, these data show that NVX-CoV2373 induces a relatively broad humoral and cellular immune response against SARS-CoV-2 in humans, and might demonstrate distinctive long-term behavior relative to mRNA vaccines.

## Methods

### Human subjects

PBMCs were obtained from the subjects in study 2019nCoV-101, a phase I/II clinical trial of NVX-CoV2373 carried out in male and female adults in Australia and the United States. Dosing was carried out under supervision of a Data and Safety Monitoring Board. Descriptions of parts I and II of this trial have been published elsewhere (1, 8). Subjects included healthy adults without evidence of preceding SARS-CoV-2 infection and ages 18 to 59 years in part I, with the addition of a 60- to 84-year-old stratum in part II. Donors of PBMC fractions for the studies reported here were selected randomly from among individuals who had adequate specimens at all 3 specified dates (baseline, 7 days after dose 1, and 7 days after dose 2) and were treated twice with 5 μg SARS-CoV-2 spike antigen plus 50 μg Matrix-M adjuvant (Novavax) at a 21-day interval, as this was the dose and regimen selected to go forward for further clinical development. PBMCs from 5 recipients of placebo were included among the study samples in a blinded fashion.

### Flow cytometry

#### T cell stimulation.

For all flow cytometry assays of stimulated T cells, cryopreserved cells were thawed by diluting them in 10 mL prewarmed complete RPMI containing 5% human AB serum (Gemini Bioproducts) in the presence of Benzonase (20 μL/10 mL, MilliporeSigma) and spun at 1200 rpm for 7 minutes. Supernatants were carefully removed by pipetting and cells were resuspended in warm medium, counted, and apportioned for assays.

#### AIM assay.

AIM assays were conducted as previously described (9, 30, 31). Cells were cultured for 24 hours in the presence of SARS-CoV-2–specific MPs (1 μg/mL) in 96-well U-bottom plates at 1 × 10^6^ PBMCs per well in complete RPMI containing 5% human AB Serum. Prior to addition of peptide MPs, cells were blocked at 37°C for 15 minutes with 0.5 μg/mL anti-CD40 mAb (Miltenyi Biotec). A stimulation with an equimolar amount of DMSO was performed as negative control; staphylococcal enterotoxin B (SEB, 1 μg/mL) and stimulation with a combined CD4^+^ and CD8^+^ T cell cytomegalovirus MP (CMV, 1 μg/mL) were included as positive controls. After incubation, cells were washed and stained with Fixable Live/Dead Blue stain (Thermo Fisher Scientific, L23105) in the presence of anti-CD16/anti-CD32 human FC block (Biolegend, 422302) at room temperature for 15 minutes. Cells were washed and then stained for surface markers CD3 (BUV395, BD Biosciences, 563546), CD4 (cFluor b548, Cytek Biosciences, R7-20043), CD8 (BUV805, BD Biosciences, 612889), ICOS (BUV563, BD Biosciences, 741421), PD-1 (BV785, Biolegend, 329930), CD16 (BV510, Biolegend, 302048), CD14 (BV510, Biolegend, 367124), CD20 (BV510, Biolegend, 302340), CD45RA (BV570, Biolegend, 304132), CCR7 (BV711, Biolegend, 353228), CXCR5 (BV421, Biolegend, 356920), CD69 (FITC, Biolegend, 310904), CD137 (BUV737, BD Biosciences, 741861), OX40 (APC, Biolegend, 350008), CD40L (PE-Dazzle, Biolegend, 310840), PDL-1 (PE, Biolegend, 329706), CXCR3 (BV605, Biolegend, 353728), and CCR6 (BUV496, BD Biosciences, 612948) for 30 minutes at 4°C. Samples were washed and immediately acquired on a Cytek Aurora 5-laser spectral analyzer (Cytek Biosciences). Data were analyzed in FlowJo, version 10 (BD Biosciences). AIM^+^ gates were drawn relative to the unstimulated condition for each donor. Stimulation index was calculated as the background-subtracted signal in the test condition divided by the average response detected in the DMSO negative control wells for that sample. Values were set to the LOD for the assay if the stimulation index was less than 2. Poor-quality samples were identified as samples with an SEB response less than 50% of the median SEB response for all samples, and were excluded from downstream analyses. The LOQ was calculated as the geometric mean of all sample DMSO wells multiplied by the geometric SD. The percentage responders was calculated as responses greater than or equal to the LOQ divided by the total samples in the group.

#### ICS assay.

As for the AIM assay, cells were blocked at 37°C for 15 minutes with 0.5 μg/mL anti-CD40 mAb before the addition of MPs. PBMCs were cultured in the presence of SARS-CoV-2 MPs (1 μg/mL) for 24 hours at 37°C. In addition, PBMCs were incubated with an equimolar amount of DMSO as a negative control and also CMV MP (1 μg/mL) as a positive control. After 24 hours, 1 μL/mL of both Golgi-Plug and Golgi-Stop (BD Biosciences) were added to the culture for an additional 4 hours along with AIM marker antibodies against CD69 (BV605, BD Biosciences, 562989), CD137 (PE-Cy5, Biolegend, 309808), and CD40L (PerCP-ef710, Thermo Fisher Scientific, 46-1548-42). After incubation, cells were washed and stained with Fixable Live/Dead Blue stain (Thermo Fisher Scientific, L23105) in the presence of anti-CD16/anti-CD32 human FC block (Biolegend, 422302) at room temperature for 15 minutes. Cells were washed and stained for additional surface markers CD8 (BUV805, BD Biosciences, 612889), CD16 (BV510, Biolegend 302048), CD14 (BV510, Biolegend, 367124), CD20 (BV510, Biolegend,302340), CD3 (BUV395, BD Biosciences, 563546), CD45RA (BV570, Biolegend, 304132), CCR7 (PE-Cy7, Biolegend, 353226), and CD4 (cFluor b548, Cytek Biosciences, R7-20043) for 30 minutes at 4°C in the dark. Cells were then washed and fixed with 1% paraformaldehyde (Sigma-Aldrich). Subsequently, cells were permeabilized and stained with intracellular antibodies against IFN-γ (FITC, eBioscience, 11-7319-82), IL-4 (BUV737, BD Biosciences, 612835), IL-17A (BV785, Biolegend, 512338), IL-2 (BB700, BD Biosciences, 566405), TNF-α (eFluor450, eBioscience, 48-7349-42), IL-10 (eFluor450, eBioscience, 48-7349-42), and granzyme B (AF647, BD Biosciences, 560212) for 30 minutes at room temperature in the dark. Samples were washed and acquired on a Cytek Aurora 5-laser spectral analyzer (Cytek Biosciences). Data were analyzed in FlowJo, version 10. ICS^+^ gates were drawn relative to the unstimulated condition for each donor. LOQ and percentage responders were calculated as described above. Boolean cytokine expression data were input into SPICE, an open-source graphing tool for complex data sets (32), to create pie charts in Supplemental Figures 2 and 3.

### Anti-spike IgG ELISAs

Recombinant SARS-CoV-2 (rSARS-CoV-2) spike protein was immobilized onto the surface of 96-well microtiter plates by direct adsorption at 2°C to 8°C, followed by washing and blocking. Diluted reference standard (2-fold dilution series of 12 dilutions starting 1:1000) and human serum samples (3-fold dilution series of 12 dilutions) in assay buffer were then added in duplicate (100 μL per well) to the spike protein–coated wells and specific antibodies were allowed to complex with the coated antigen for 2 hours ± 10 minutes at 24°C ± 2°C. After washing, IgG bound to the rSARS-CoV-2 spike protein was detected using a horseradish peroxidase–conjugated (HRP-conjugated) goat anti-human IgG antibody (Southern Biotech) incubated for 1 hour ± 10 minutes at 24°C ± 2°C. After further washing, a colorimetric signal was generated by addition of 100 μL per well of 3,3′,5,5′-tetramethylbenzidine (TMB) substrate for 10 minutes ± 2 minutes at 24°C ± 2°C. The TMB reaction was stopped with 100 μL per well of TMB Stop solution, and absorbance was measured at 450 nm. Anti–rSARS-CoV-2 spike protein IgG antibody level in clinical serum samples was quantitated in ELISA units, EU/mL, by comparison to a reference standard curve. The results were analyzed by SoftMax Pro software (Molecular Devices) using a 4-parameter logistic (4-PL) curve fit. Each assay run included control plates consisting of positive and negative controls.

### hACE2 binding inhibition assay

rSARS-CoV-2 spike protein was immobilized onto the surface of 96-well microtiter plates by direct adsorption at 2°C to 8°C, followed by washing and blocking. Serial dilutions of human serum samples, including assay quality controls (QCs), were then added to the spike-coated wells and any molecules that could bind to the spike protein, presumptively primarily spike-specific antibodies, were allowed to complex with the immobilized spike protein (for 1 hour at 24°C ± 2°C). After a plate-washing step, a fixed concentration of human ACE2 receptor (hACE2) with a polyhistidine tag (His-Tag) (SinoBiological) was added to the plate for incubation (1 hour at 24°C ± 2°C) during which the hACE2 bound to the spike protein residues with binding sites not obstructed by bound antibody. After washing, the hACE2 receptor bound to the spike protein was then detected using a mouse anti–His-Tag HRP conjugate (Southern Biotech) and a colorimetric signal generated by the addition of TMB substrate. The amount of bound hACE2 detected was inversely proportional to the amount hACE2 binding inhibitors (antibodies) in human serum; inhibitory activity is reported as 50% inhibitory titers based on a 4-PL curve fit (SoftMax Pro software).

### SARS-CoV-2 microneutralization

Microneutralization assays were performed at the University of Maryland in the laboratory of Matthew Frieman using heat-inactivated (56°C for 30 minutes) sera. Samples were diluted in duplicate to a base dilution of 1:5 or 1:10, followed by 11 × 1:2 serial dilutions in DMEM (Quality Biologicals) supplemented with 10% fetal bovine serum (heat inactivated, Sigma-Aldrich), 1% penicillin/streptomycin) (Gemini Bio-Products) and 2 mM L-glutamine (Gibco), resulting in 100 μL per well. The dilution plates were then transferred to a BSL-3 environment and 100 μL of prediluted SARS-CoV-2 inoculum was added to result in a multiplicity of infection (MOI) of 0.01 upon transfer to 96-well plates. Virus-only and mock-infection wells were included in each assay. After incubation of the mixtures at 37°C and 5% CO_2_ for 1 hour, the mixtures were transferred to 96-well plates with confluent VeroE6 cells. The plates were then further incubated at 37°C and 5% CO_2_ for 72 hours, followed by examination for cytopathic effect (CPE). The first dilution to show CPE is reported as the minimum dilution required to inhibit (neutralize) greater than 99% of the inoculum of SARS-CoV-2 tested.

### Statistics

Data were analyzed using FlowJo 10.7.1. Statistical analyses were performed in GraphPad Prism 9.2. The statistical details of the experiments are provided in the respective figure legends. Data plotted in linear scale are expressed as means ± SEM. Data plotted in logarithmic scales are expressed as geometric means ± geometric SDs. Mann-Whitney *U* or Wilcoxon’s test was applied for unpaired or paired comparisons, respectively. A *P* value of 0.05 was used to determine significance of differences.

### Study approval

Samples were acquired as part of USDA clinical trial NCT04368988. The trial protocol was approved by the Alfred Hospital Ethics Committee (Melbourne, VIC, Australia) and Advarra Central Institutional Review Board (Columbia, Maryland, USA) and was performed in accordance with the International Conference on Harmonisation, Good Clinical Practice guidelines.

## Author contributions

LF, GG, and SC conceptualized the study. CRM, CK, JM, JP, MZ, SCC, AS, DW, and SC developed the methodology. CRM, CK, JP, MZ, LF, GG, AS, and SC carried out formal analyses of the data. CRM, AS, LF, GG, and SC conducted the investigation. GG, AS, and SC acquired funding. CRM, LF, GG, and SC wrote the manuscript. LF, GG, and SC supervised the study.

## Supplementary Material

Supplemental data

## Version 1 08/09/2022

In-Press Preview

## Version 2 10/03/2022

Electronic publication
