# Supplementary material for: NVX-CoV2373 vaccination induces functional SARS-CoV-2–specific CD4+ and CD8+ T cell responses
Source: J Clin Invest. 2022 Oct 3;132(19):e160898. doi: 10.1172/JCI160898 (PMC9525112; doi:10.1172/JCI160898)
Supplement: Supplemental data [file jci-132-160898-s078.pdf]

SUPPLEMENTARY FIGURES

Figure S1

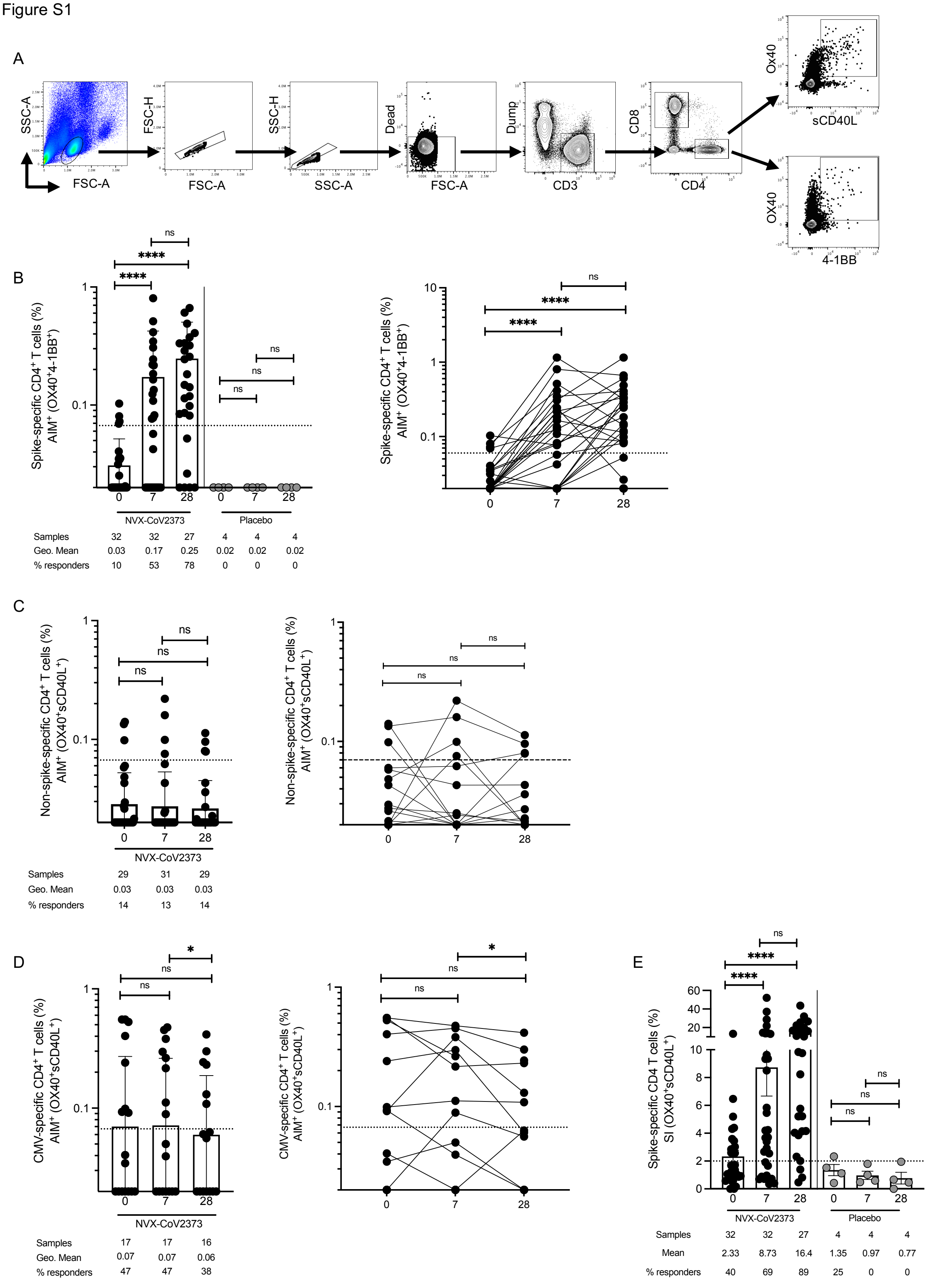

Figure S1. Spike-specific CD4$^+$ T cells following NVX-CoV2373 vaccination. **(A)** FACS gating strategy used to define AIM$^+$CD4$^+$ T cells at D0, D7 and D28 post-vaccination. **(B)** Spike-specific OX40$^+$4-1BB$^+$ AIM$^+$ CD4$^+$ T cell responses in vaccinees (black dots) and placebo controls (grey dots), graphed as both grouped and paired comparisons for the vaccine cohort (**C)** SARS-CoV-2 non-spike-specific OX40$^+$CD40L$^+$ AIM$^+$ CD4$^+$ T cell responses in vaccinees at D0, 7 and 28 post-vaccination. **(D)** CMV class I/II MP-specific OX40$^+$CD40L$^+$ AIM$^+$ CD4$^+$ T cell responses in vaccinees at D0, 7 and 28 post-vaccination. **(E)** Stimulation indices for OX40$^+$CD40L$^+$ CD4$^+$ AIM$^+$ T cells in vaccinees and controls at D0, D7 and D28 post-vaccination. Dotted line indicates LOQ, and is calculated as the geometric mean of all sample DMSO wells multiplied by the geometric SD factor. % responders is calculated as responses ≥ LOQ divided by the total samples in the group. Paired data were analyzed by Wilcoxon signed rank test. Log data shown with geometric mean ± geometric SD. Linear data shown with mean ± SEM. * $p<0.05$, ** $p<0.01$, *** $p<0.001$, ****$p<0.0001$.

Figure S2

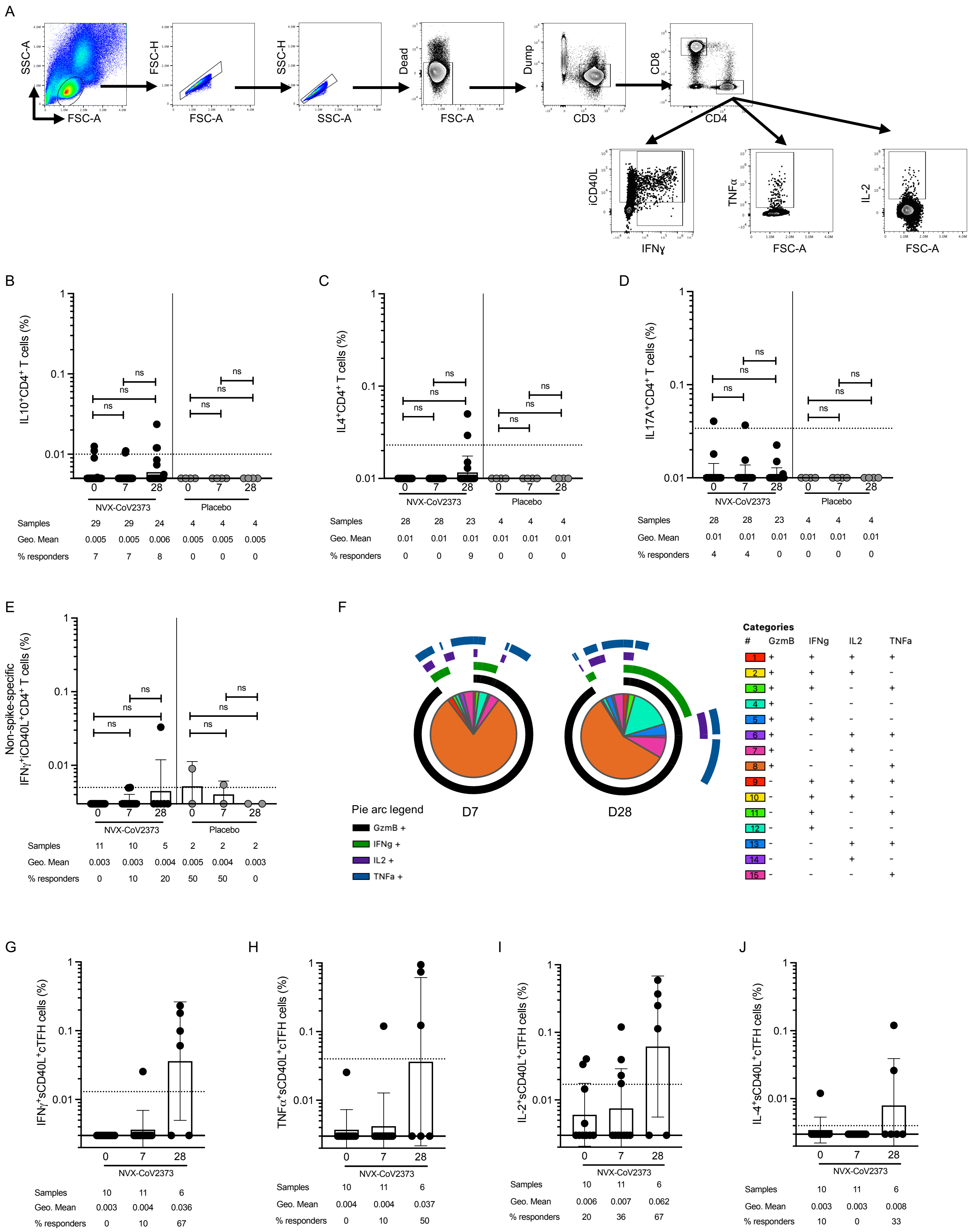
A
SSC-A
FSC-A
FSC-H
FSC-A
SSC-H
SSC-A
Dead
FSC-A
Dump
CD3
CD8
CD4
iCD40L
IFNγ
TNFα
FSC-A
IL-2
FSC-A
B
IL10+CD4+ T cells (%)
NVX-CoV2373
Placebo
Samples 29 29 24 4 4 4
Geo. Mean 0.005 0.005 0.006 0.005 0.005 0.005
% responders 7 7 8 0 0 0
C
IL4+CD4+ T cells (%)
NVX-CoV2373
Placebo
Samples 28 28 23 4 4 4
Geo. Mean 0.01 0.01 0.01 0.01 0.01 0.01
% responders 0 0 9 0 0 0
D
IL17A+CD4+ T cells (%)
NVX-CoV2373
Placebo
Samples 28 28 23 4 4 4
Geo. Mean 0.01 0.01 0.01 0.01 0.01 0.01
% responders 4 4 0 0 0 0
E
Non-spike-specific IFNγ+iCD40L+CD4+ T cells (%)
NVX-CoV2373
Placebo
Samples 11 10 5 2 2 2
Geo. Mean 0.003 0.003 0.004 0.005 0.004 0.003
% responders 0 10 20 50 50 0
F
D7
D28
Pie arc legend
GzmB +
IFNg +
IL2 +
TNFa +
Categories
GzmB IFNg IL2 TNFa
1 + + + +
2 + + + -
3 + + - +
4 + - - -
5 + + - -
6 + - + +
7 + - + -
8 + - - +
9 - + + +
10 - + + -
11 - + - +
12 - + - -
13 - - + +
14 - - + -
15 - - - +
G
IFNγ+sCD40L+cTFH cells (%)
NVX-CoV2373
Samples 10 11 6
Geo. Mean 0.003 0.004 0.036
% responders 0 10 67
H
TNFα+sCD40L+cTFH cells (%)
NVX-CoV2373
Samples 10 11 6
Geo. Mean 0.004 0.004 0.037
% responders 0 10 50
I
IL-2+sCD40L+cTFH cells (%)
NVX-CoV2373
Samples 10 11 6
Geo. Mean 0.006 0.007 0.062
% responders 20 36 67
J
IL-4+sCD40L+cTFH cells (%)
NVX-CoV2373
Samples 10 11 6
Geo. Mean 0.003 0.003 0.008
% responders 10 0 33

Figure S2. Cytokine-producing spike-specific $CD4^+$ T cell responses following NVX-CoV2373 vaccination. **(A)** FACS gating strategy used to define $cytokine^+$ $CD4^+$ T cells at D0, D7 and D28 post-vaccination. Proportion of **(B)** $IL10^+$, **(C)** $IL4^+$ and **(D)** $IL17A^+$ spike-specific CD4 T cells detected following peptide stimulation. **(E)** $IFN\gamma^+CD40L^+$ $CD4^+$ T cells measured in response to SARS-CoV-2 Remainder MP stimulation. **(F)** Pie charts depicting the proportion of spike-specific $CD4^+$ T cells exhibiting different combination of cytokines and/or effector molecules at D7 and D28 post-1st immunization. **(G)** $sCD40L^+IFN\gamma^+$, **(H)** $sCD40L^+TNF\alpha^+$, **(I)** $sCD40L^+IL\text{-}2^+$, and **(J)** $sCD40L^+IL\text{-}4^+$ cTFH cells detected following peptide stimulation (cTFH cells defined as $CXCR5^+CD4^+$ T cells). Dotted line indicates LOQ, and is calculated as the geometric mean of all sample DMSO wells multiplied by the geometric SD factor. % responders is calculated as responses ≥ LOQ divided by the total samples in the group. Paired data were analyzed by Wilcoxon signed rank test. Data shown with geometric mean ± geometric SD. * $p<0.05$, ** $p<0.01$, *** $p<0.001$, ****$p<0.0001$. Boolean data were input into SPICE, an open-source graphing tool for complex data sets (32), to create pie graphs.

Figure S3

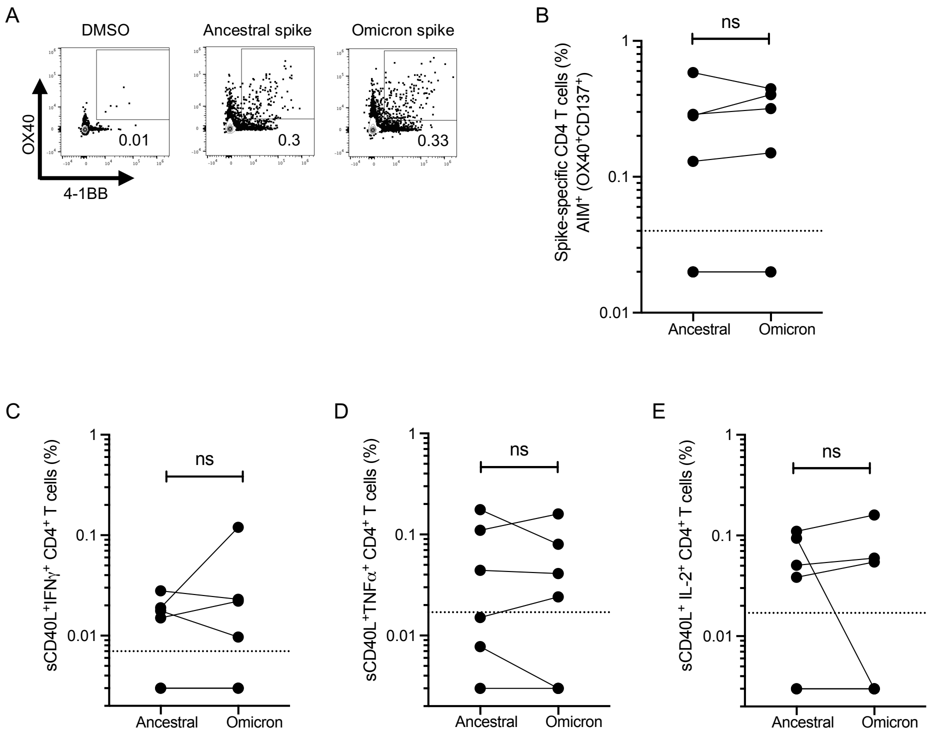

Figure S3. Ancestral and Omicron spike-specific $CD4^+$ T cells responses induced by NVX-CoV2373 vaccination. **(A)** Representative FACS gating of $OX40^+CD137^+$ $CD4^+$ T cells following stimulation with Ancestral or Omicron SARS-2 spike MP. **(B)** Paired AIM^+ $CD4^+$ **(C)** $sCD40L^+IFN\gamma^+$ **(D)** $sCD40L^+TNF\alpha^+$, and **(E)** $sCD40L^+IL\text{-}2^+$ $CD4^+$ T cells one week post-2nd immunization and stimulated with either Ancestral or Omicron SARS-2 spike MP. Dotted line indicates LOQ, and is calculated as the geometric mean of all sample DMSO wells multiplied by the geometric SD factor. Paired data were analyzed by Wilcoxon signed rank test. Data shown with geometric mean ± geometric SD. * $p<0.05$, ** $p<0.01$, *** $p<0.001$, ****$p<0.0001$.

Figure S4

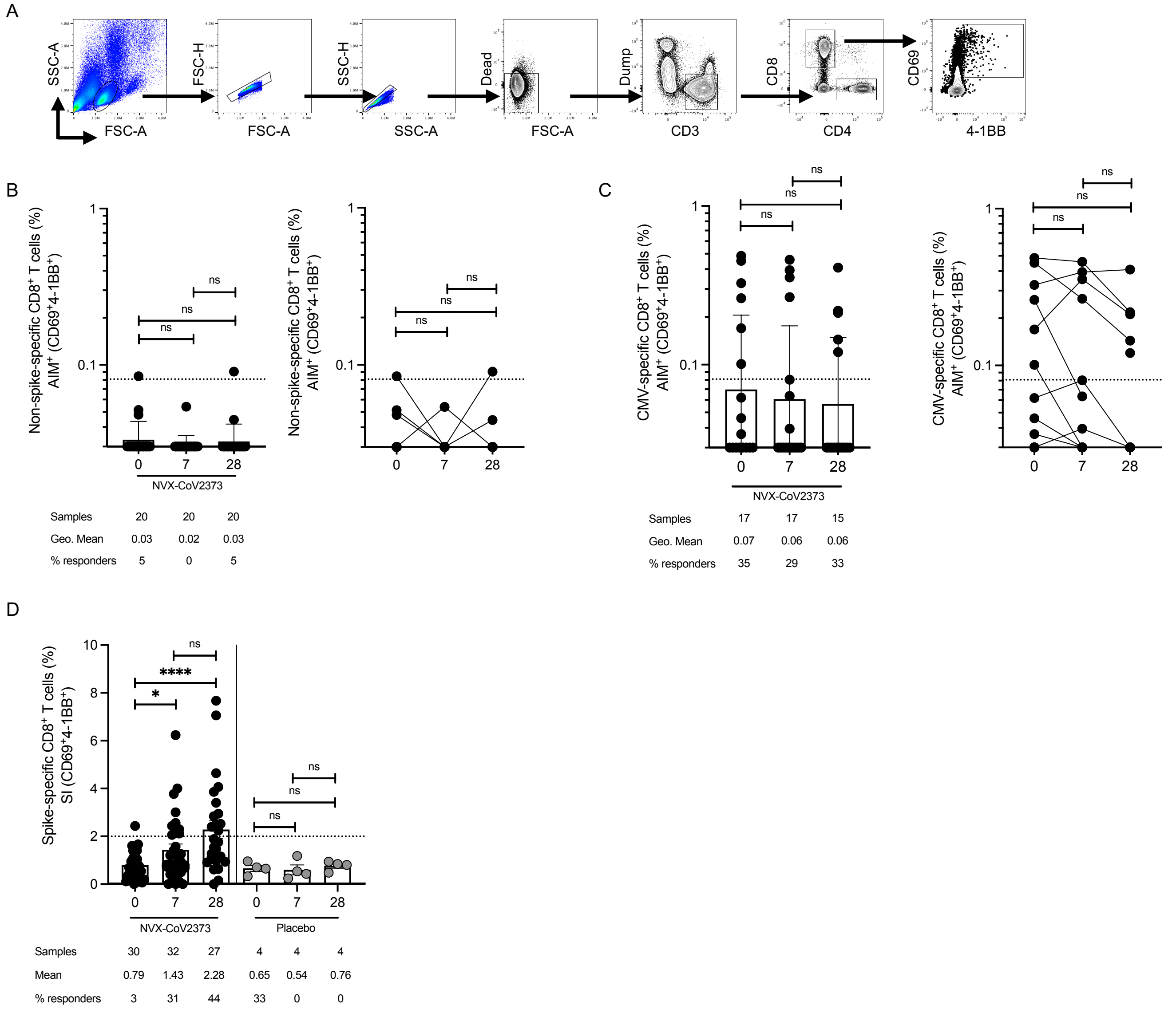

Figure S4. Spike-specific CD8⁺ T cells are induced following NVX-CoV2373 vaccination**.** **(A)** FACS gating strategy used to define AIM⁺ CD8⁺ T cells at D0, D7 and D28 post-vaccination. **(B)** SARS-CoV-2 Remainder MP-specific CD69⁺4-1BB⁺ AIM⁺ CD8⁺ T cell responses in vaccinees at D0, 7 and 28 post-vaccination. **(C)** CMV class I/II MP-specific CD69⁺4-1BB⁺ AIM⁺ CD8⁺ T cell responses in vaccinees at D0, 7 and 28 post-vaccination. **(D)** Stimulation indices for CD69⁺4-1BB⁺ CD8⁺ AIM⁺ T cells in vaccinees and controls at D0, D7 and D28 post-vaccination. Paired data were analyzed by Wilcoxon signed rank test. Log data shown with geometric mean ± geometric SD. Linear data shown with mean ± SEM. * $p<0.05$, ** $p<0.01$, *** $p<0.001$, ****$p<0.0001$.

Figure S5

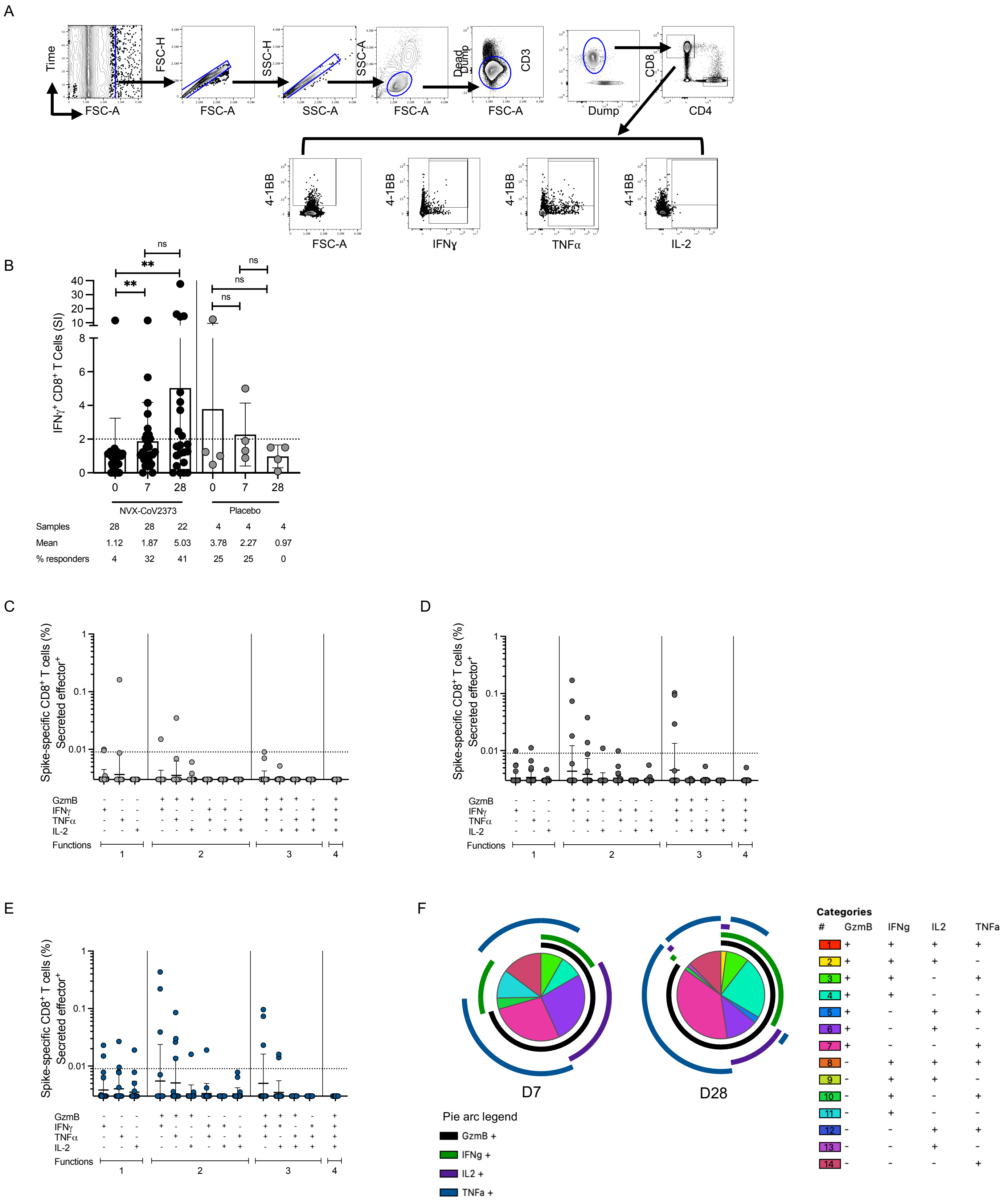
A
Time
FSC-A
FSC-H
SSC-H
SSC-A
Dead Dump
CD3
Dump
CD8
CD4
4-1BB
IFNγ
TNFα
IL-2
B
IFNγ+ CD8+ T Cells (SI)
**
ns
NVX-CoV2373
Placebo
Samples 28 28 22 4 4 4
Mean 1.12 1.87 5.03 3.78 2.27 0.97
% responders 4 32 41 25 25 0
C
D
E
Spike-specific CD8+ T cells (%) Secreted effector+
GzmB
IFNγ
TNFα
IL-2
Functions
F
D7
D28
Pie arc legend
GzmB +
IFNg +
IL2 +
TNFa +
Categories
GzmB IFNg IL2 TNFa
1 + + + +
2 + + + -
3 + + - +
4 + + - -
5 + - + +
6 + - + -
7 + - - +
8 - + + +
9 - + + -
10 - + - +
11 - + - -
12 - - + +
13 - - + -
14 - - - +

Figure S5. $CD8^+$ T cell cytokine responses induced by NVX-CoV2373 vaccination. **(A)** Gating strategy used to identify cytokine-producing $CD8^+$ T cells. **(B)** $IFN\gamma^+CD8^+$ T cells measured in response to SARS-CoV-2 non-spike MP stimulation. Predominant multifunctional profiles of spike-specific $CD8^+$ T cells with one, two, three or four functions were analyzed at **(C)** D0, **(D)** D7, and **(E)** D28 post vaccination. Dotted line indicates LOQ for the assay, and is calculated as the geometric mean of all sample DMSO wells multiplied by the geometric SD factor. % responders is calculated as responses ≥ LOQ divided by the total samples in the group. **(F)** Pie charts depicting the proportion of spike-specific $CD4^+$ T cells exhibiting different combination of cytokines and/or effector molecules at D7 and D28 post-1st immunization. Paired data were analyzed by Wilcoxon signed rank test. Data shown with geometric mean ± geometric SD. * $p<0.05$, ** $p<0.01$, *** $p<0.001$, ****$p<0.0001$. Boolean data were input into SPICE, an open-source graphing tool for complex data sets (32), to create pie graphs.

Figure S6

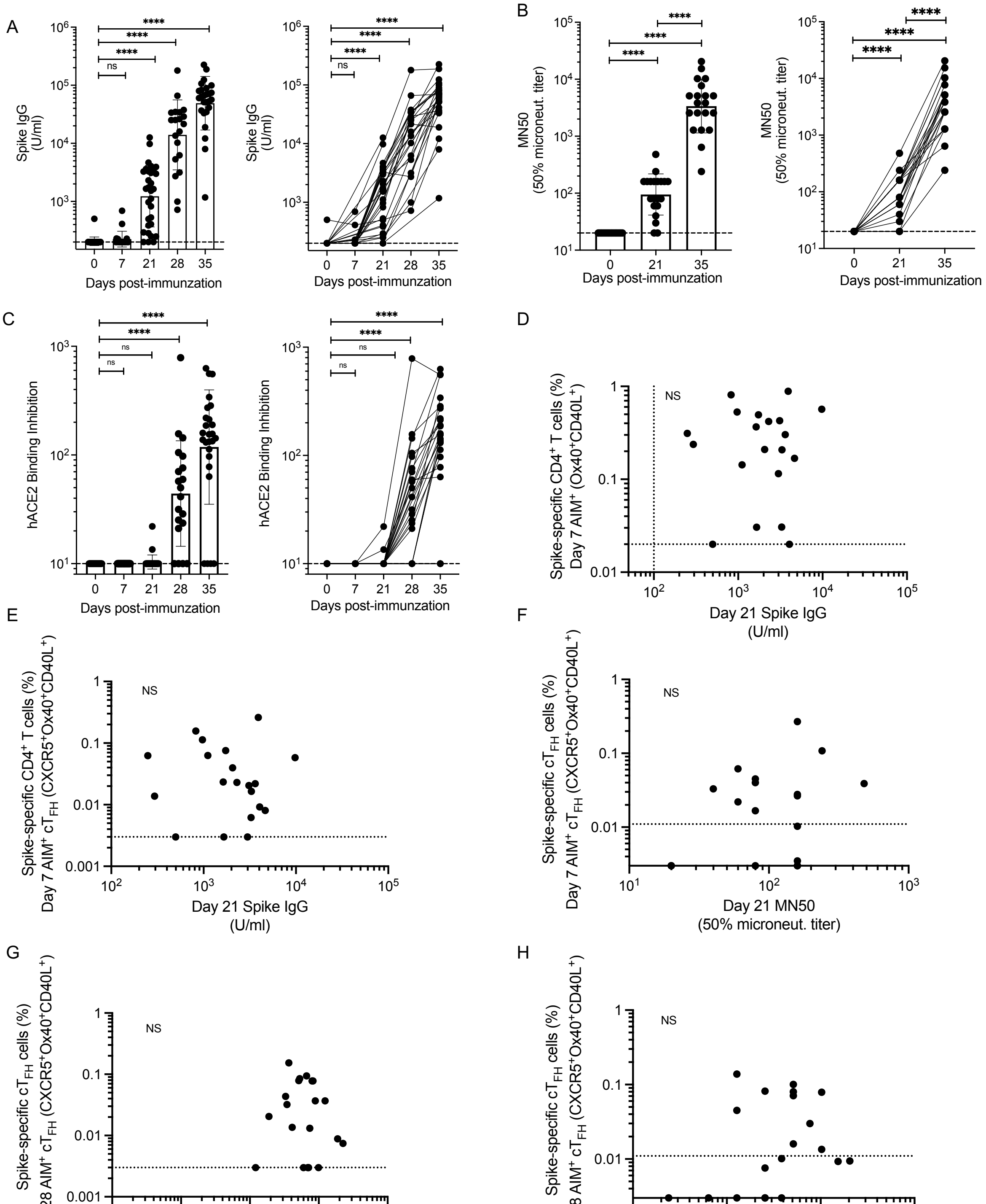

A
Spike IgG (U/ml)
Days post-immunzation
ns
****
B
MN50 (50% microneut. titer)
Days post-immunzation
Days post-immunization
C
hACE2 Binding Inhibition
D
Spike-specific CD4+ T cells (%) Day 7 AIM+ (Ox40+CD40L+)
Day 21 Spike IgG (U/ml)
NS
E
Spike-specific CD4+ T cells (%) Day 7 AIM+ cTFH (CXCR5+Ox40+CD40L+)
F
Spike-specific cTFH cells (%) Day 7 AIM+ cTFH (CXCR5+Ox40+CD40L+)
Day 21 MN50 (50% microneut. titer)
G
Spike-specific cTFH cells (%) Day 28 AIM+ cTFH (CXCR5+Ox40+CD40L+)
Day 35 Spike IgG (U/ml)
H
Day 35 MN50 (50% microneutralization titer)

Figure S6. Association between T cells and antibody responses following NVX-CoV2373 vaccination. **(A)** Spike IgG titers, **(B)** SARS-CoV-2 neutralization titers, and **(C)** hACE2 binding inhibition measured in vaccinees at D0, 7, and 28 post-immunization. **(D)** Correlation of D7 SARS-CoV-2 Spike-specific AIM$^+$ CD4$^+$ T cells and D21 spike-specific IgG. Spike IgG and neutralizing antibody correlations with AIM$^+$ cT$_{FH}$ cells at **(E, F)** D7 post-1st, or **(G, H)** D7 post-2nd immunization. Paired data were analyzed by Wilcoxon signed rank test **(A-C)** and Spearman correlation **(D-H)**. Data shown with geometric mean $\pm$ geometric SD. * $p<0.05$, ** $p<0.01$, *** $p<0.001$, ****$p<0.0001$.

Figure S7

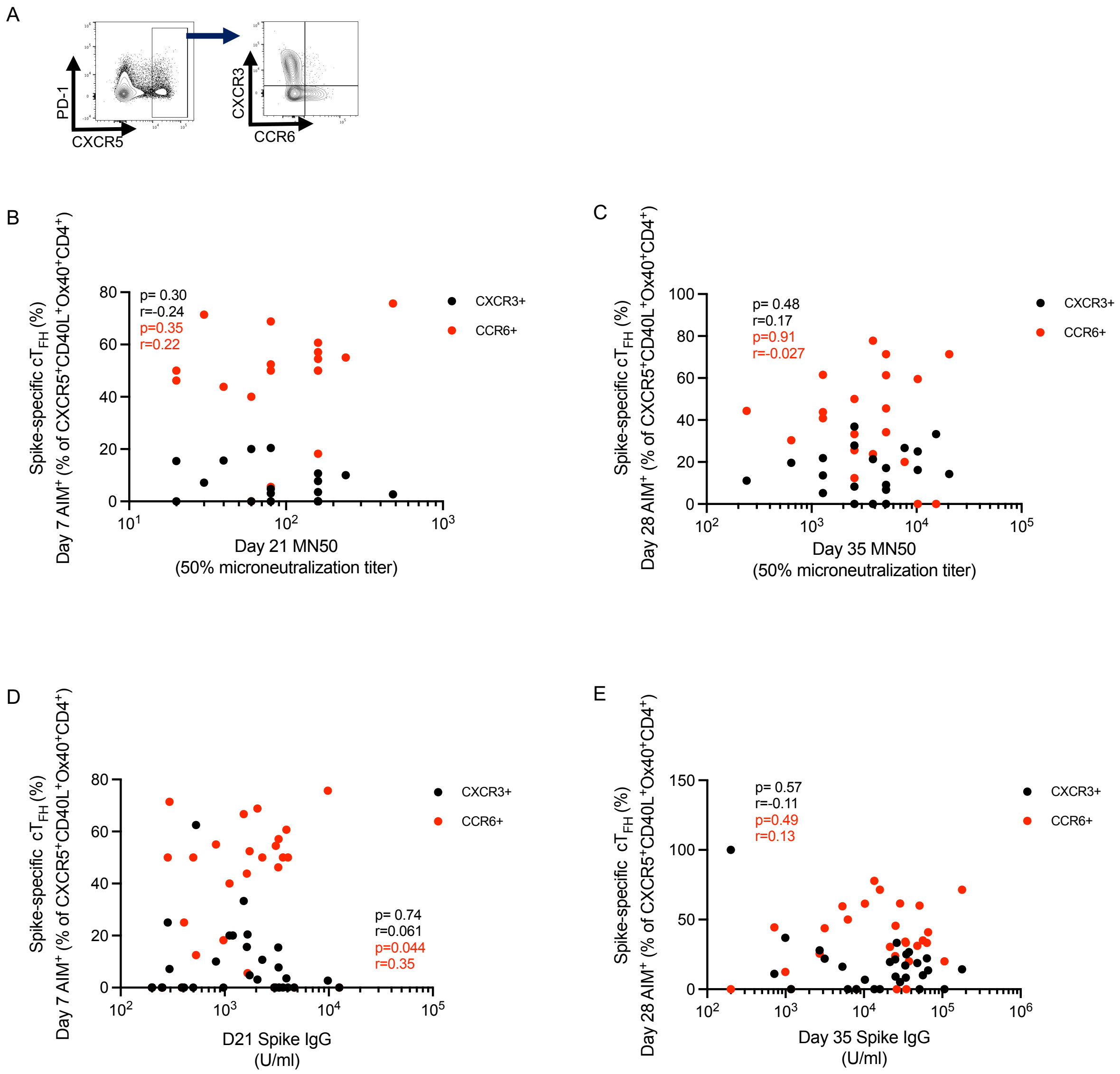

Figure S7. Association between antibody responses and cT_{FH} cell subsets following NVX-CoV2373 vaccination. **(A)** Representative FACS gating of CXCR3- and CCR6- expressing cTFH cells. **(B)** Correlation of $CXCR3^+$ $CCR6^+$ cTFH 7 days post-1st immunization and d21 post-1st immunization microneutralization titers. **(C)** Correlation of $CXCR3^+$ $CCR6^+$ cTFH 7 days post-2nd immunization and d14 post-2nd immunization microneutralization titers. **(D)** Correlation of $CXCR3^+$ $CCR6^+$ cTFH 7 days post-1st immunization and d21 post-1st immunization SARS-2 spike IgG titers. **(E)** Correlation of $CXCR3^+$ $CCR6^+$ cTFH 7 days post-2nd immunization and d14 post-2nd immunization SARS-2 spike IgG titers. $CXCR3^+CXCR5^+CD4^+$ T cells are shown in black and $CCR6^+CXCR5^+CD4^+$ T cells are shown in red. Data were analyzed by Spearman correlation.

Figure S8

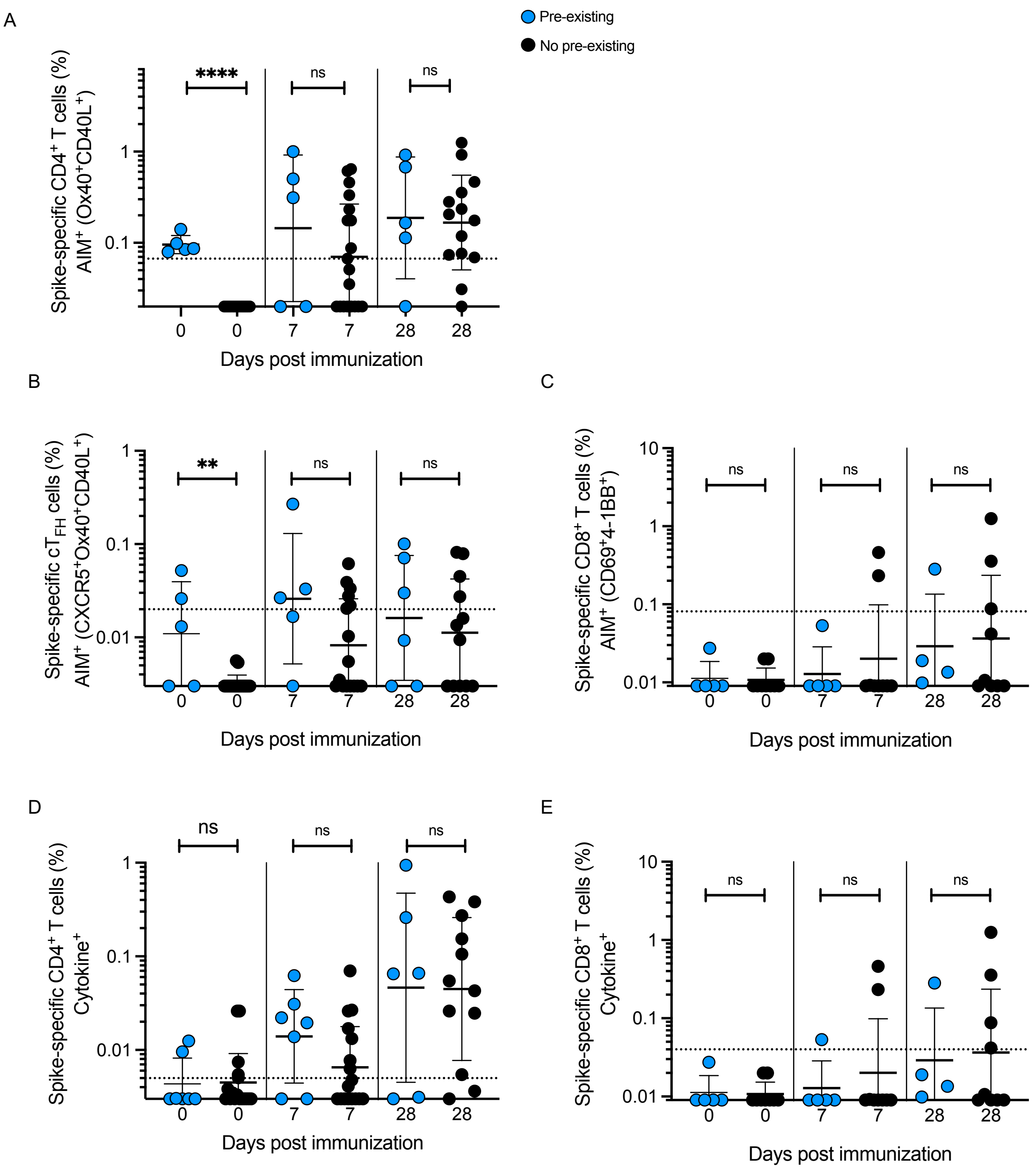
A
Pre-existing
No pre-existing
Spike-specific CD4+ T cells (%)
AIM+ (Ox40+CD40L+)
****
ns
ns
Days post immunization
B
Spike-specific cTFH cells (%)
AIM+ (CXCR5+Ox40+CD40L+)
**
ns
ns
Days post immunization
C
Spike-specific CD8+ T cells (%)
AIM+ (CD69+4-1BB+)
ns
ns
ns
Days post immunization
D
Spike-specific CD4+ T cells (%)
Cytokine+
ns
ns
ns
Days post immunization
E
Spike-specific CD8+ T cells (%)
Cytokine+
ns
ns
ns
Days post immunization

Figure S8. Pre-existing T cell immunity does not impact NVX-CoV2373 vaccine responses. **A)** Spike-specific $CD4^+$ AIM^+ T cells at baseline and post-vaccination grouped according to donors with $CD4^+AIM^+$ T cell responses at D0 above the LOS for the assay (blue dots) or no detectable $CD4^+$ AIM^+ T cell responses at D0 (black dots). Effect of high pre-existing $CD4^+$ AIM^+ T cells on **(B)** spike specific AIM^+ cT_{FH} cells, **(C)** spike-specific AIM^+ $CD8^+$ T cells, **(D)** total Spike-specific $CD4^+$ T cell and **(E)** spike-specific $CD8^+$ T cell cytokine production. Dotted line indicates LOQ for the assay, and is calculated as the geometric mean of all sample DMSO wells multiplied by the geometric SD factor. Data were analyzed by Mann-Whitney test. Data shown with geometric mean ± geometric SD. * $p<0.05$, ** $p<0.01$, *** $p<0.001$, ****$p<0.0001$.
